# OLEAtool: An open-source software for morphopalynological research in *Olea europaea* L. pollen

**DOI:** 10.12688/openreseurope.15309.1

**Published:** 2023-02-08

**Authors:** Gabriel Servera-Vives, Cristina Ricucci, Grant Snitker

**Affiliations:** 1 ArqueoUIB, Department of Historical Sciences and Theory of Art, University of the Balearic Islands, Carretera de Valldemossa Km 7,5, CP07122 Palma, Mallorca, Spain; 2 Laboratory of Palynology and Palaeobotany, Department of Life Sciences, Università degli Studi di Modena e Reggio Emilia, Modena, Italy; 3 Cultural Resource Sciences, New Mexico Consortium, 4200 W. Jemez Rd. Suite 301, Los Alamos, New Mexico, 87544, USA

**Keywords:** OLEAtool, pollen morphology, ImageJ, image analysis, olive tree, open-source software, microscopy

## Abstract

In this paper we present OLEAtool, a new software tool for palynological research to facilitate morphological analysis and measurements of *Olea* pollen. OLEAtool is a macro extension for use with ImageJ, an open-access and freely available image analysis software, and was developed as a component of the OLEA-project. This larger project examines olive tree expansion and mosaic landscape formation on the Balearic Islands. Pollen analysis of both fossil and modern grains has been proven useful for characterizing cultivars and therefore an important method for studying olive tree cultivation in the Mediterranean. However, these methods still struggle with distinguishing between wild and cultivated varieties. Traditional morphological analysis of pollen grains can be a difficult and time-consuming task. However, OLEAtool dramatically increases the speed of collecting data on pollen grains, expands the number of variables an analyst can measure, and greatly enhances the replicability of morphological analysis.

## Plain language summary

Pollen plays a key role in reproduction for seed plants. Palynology is the study of pollen and spores with a wide variety of applications, such as the study of past landscapes or the characterization of agronomic cultivars. The onset of the olive tree management and the origin of its domestication in the Mediterranean still remains unclear, despite great advances in methods and interpretation over the last decades. Pollen analysis may help in identifying the distribution and historical trends of this species, but it is still difficult to distinguish between cultivated and wild varieties through pollen analysis. To shed some light into this issue, we have developed OLEAtool, a new extension for the open-source image analysis software, ImageJ. OLEAtool uses image analysis and replicable measurements to standardize and improve pollen morphology studies.

## Introduction

Distinguishing between wild and cultivated olive trees based on their pollen morphology is an important consideration for reconstructing the domestication history of this species. However, current methods do not adequately address this distinction and subsequently, tracing the long-term process of olive domestication remains an elusive task ( Beug, 2004; Messora *et al*., 2017; Ribeiro *et al*., 2012). Therefore, new research on characterizing *Olea europaea* (both wild and cultivated) pollen is needed to advance how we measure and describe olive pollen to more effectively discriminate between cultivars or varieties based on their morphology. Additionally, we recognize that morphological studies of modern olive pollen should be applicable to past pollen reconstructions, which will aid in characterizing olive cultivation history and identifying potential “historical” varieties.

The olive tree ( *Olea europaea* L.) is a major feature of current circum-Mediterranean landscapes and an essential element of modern Mediterranean agriculture. It is one of the most economically important trees in the Mediterranean basin and had significant cultural and symbolic value through history. Wild olive ( *Olea europaea* L. subsp. *europaea* var. *sylvestris*) is also common in macchia and garrigues across the Mediterranean climate region. The early management of (wild) olive trees has often been related to village development and the neolithization process in the eastern Mediterranean ( Langgut *et al*., 2019). However, there is an increasing consensus that the spread of olive orchards was likely driven by different factors in different areas, including human activities and climate ( Carrión *et al*., 2010; Mercuri *et al*., 2013; Terral *et al*., 2004). The integration data from within and outside of archaeological sites is essential to shed light on the cultivation and large-scale management of this tree. The study of olive tree domestication in the Mediterranean remains a challenging question despite recent advances from several disciplines, including DNA ( Besnard *et al*., 2013) and macro-botanical analysis ( Terral *et al*., 2021). Pollen records are also providing new lines of evidence to understand long-term olive tree cultivation history and large-scale olive management ( Langgut *et al*., 2019).

In this framework, the EU-funded OLEA-project (G.A. 895735) aims to focus on the drivers and timing of the spread of *Olea* shrubland environments as a central feature of the current Balearic landscape by combining past (from archaeological sites and non-archaeological sites) and modern records (modern analogues and high-resolution morphopalynological analysis). Among the latter, this project has a special focus on olive tree pollen grain morphology and studying pollen grains from different varieties of both cultivated and wild olive trees from Mallorca. The goal of these efforts is to propose morphotypes that can be applied to fossil pollen records and consequently trace back olive cultivation through time.

There are numerous and distinctive morphological attributes within pollen grains which are important taxonomic descriptors. These include pollen size, shape, number, position and character of apertures, and cell wall structure ( Erdtman, 1945; Erdtman, 1952; Kremp, 1965; Stanley & Linskens, 1974). The olive tree generally produces small or medium-sized pollen grains with a predominantly oblate-spheroidal shape and three apertures ( *colpi*) along the equatorial zone. The outer layer of the pollen wall ( *exine*) ranges from thick (1.75 µm) to very thick (3.5 µm) and gives rise to a reticular structure with cavities ( *lumina*) that characterizes the surface of the granule ( De Leonardis *et al*., 1995; Punt *et al*., 2007).

**Figure 1.**
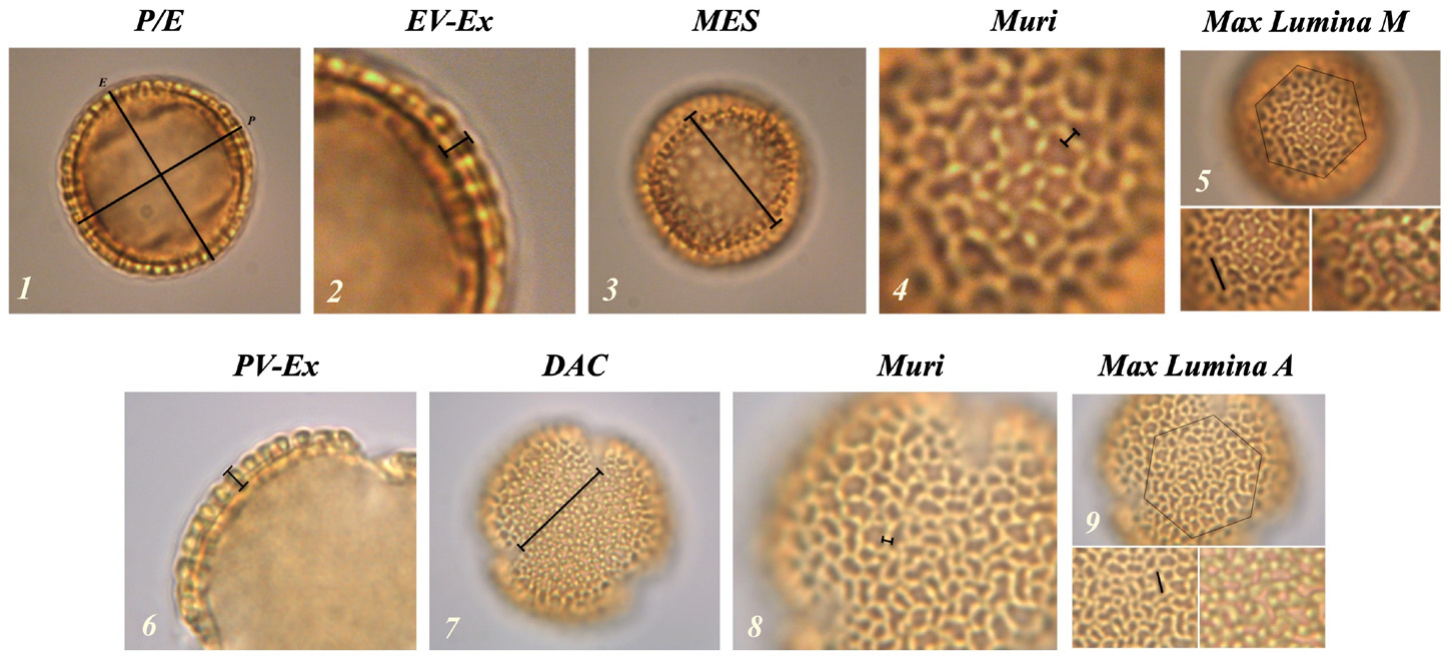
Representation of the morphological parameters in equatorial (1-5) and polar (6-9) views. (1) P / E: polar axis and equatorial diameter ratio; (2) EV-Ex: exine thickness in equatorial view; (3) MES: maximum distance between two colpi in mesocolpium; (4 and 8) Muri: reticulus thickness; (5) Lumina M: maximum length of lumina in mesocolpium; (6) PV-Ex: exine thickness in polar vision; (7) DAC: distance between the apices of two adjacent colpi; (9) Lumina A: maximum length of lumina in apocolpium.

These morphological descriptors are defined through specific parameters measurable in the two distinct views of the granule ( Figure 1). In the equatorial view, the measurable parameters are: polar axis ( *P*), equatorial diameter ( *E*), *P* / *E* ratio, exine thickness ( *EV-Ex*), maximum distance between *colpi* in *mesocolpium* ( *MES*), maximum length of *lumina* in *mesocolpium* ( *Lumina M*) and reticulum thickness ( *Muri*). In the polar view the following measurements can be observed: distance between the apices of two adjacent *colpi* in *apocolpium* ( *DAC*), exine thickness ( *PV-Ex*), reticulum thickness ( *Muri*) and maximum length of *lumina* in *apocolpium* ( *Lumina A*).

Measuring these parameters in thousands of granules is a slow and laborious process. Consequently, researchers are beginning to adopt automated techniques or digital methods meant to increase the speed and accuracy of measuring pollen grain morphology. However, studies using these techniques rarely publish information on the software configurations used to measure pollen grains, or they rely on proprietary software protected by paywalls. All these factors greatly inhibit the standardization, comparability, and reproducibility of the data and analyses such studies produce (see Daood *et al*., 2016; Khanzhina *et al*., 2018; Sevillano & Aznarte, 2018 for examples).

In response to these limitations, there is a growing trend in which funding sources and institutional research standards expect compliance with the FAIR principles (Findable, Accessible, Interoperable, and Reusable). This framework is also linked to the development of Open Science, in which the analytical procedures, data management plans and software configurations are presented transparently and available to the entire scientific community to encourage access, equity and research reproducibility ( Bartling & Friesike, 2014; Marwick *et al*., 2017). Using open-access and open-source software, in addition to publishing code and detailed user guides, supports these Open Science principles.

In this context, we present OLEAtool, an open-access macro extension for ImageJ, which provides standardized tools for digital data collection of *Olea* pollen morphological metrics. ImageJ is a free, open-source image and video analysis program originally developed in the 1990’s by the United States National Institutes of Health for processing medical imagery ( Schneider *et al*., 2012). In the years since, ImageJ has been adapted to multidisciplinary scientific applications across a diverse community of open-access researchers and practitioners. OLEAtool continues to expand the range of applications for ImageJ by including tools specifically tailored for morphopalynological data collection and analyses. By creating OLEAtool within the larger ImageJ open-science ecosystem, we are ensuring our data collection procedures are open and accessible to all.

## Methods

### Implementation

**Figure 2.**
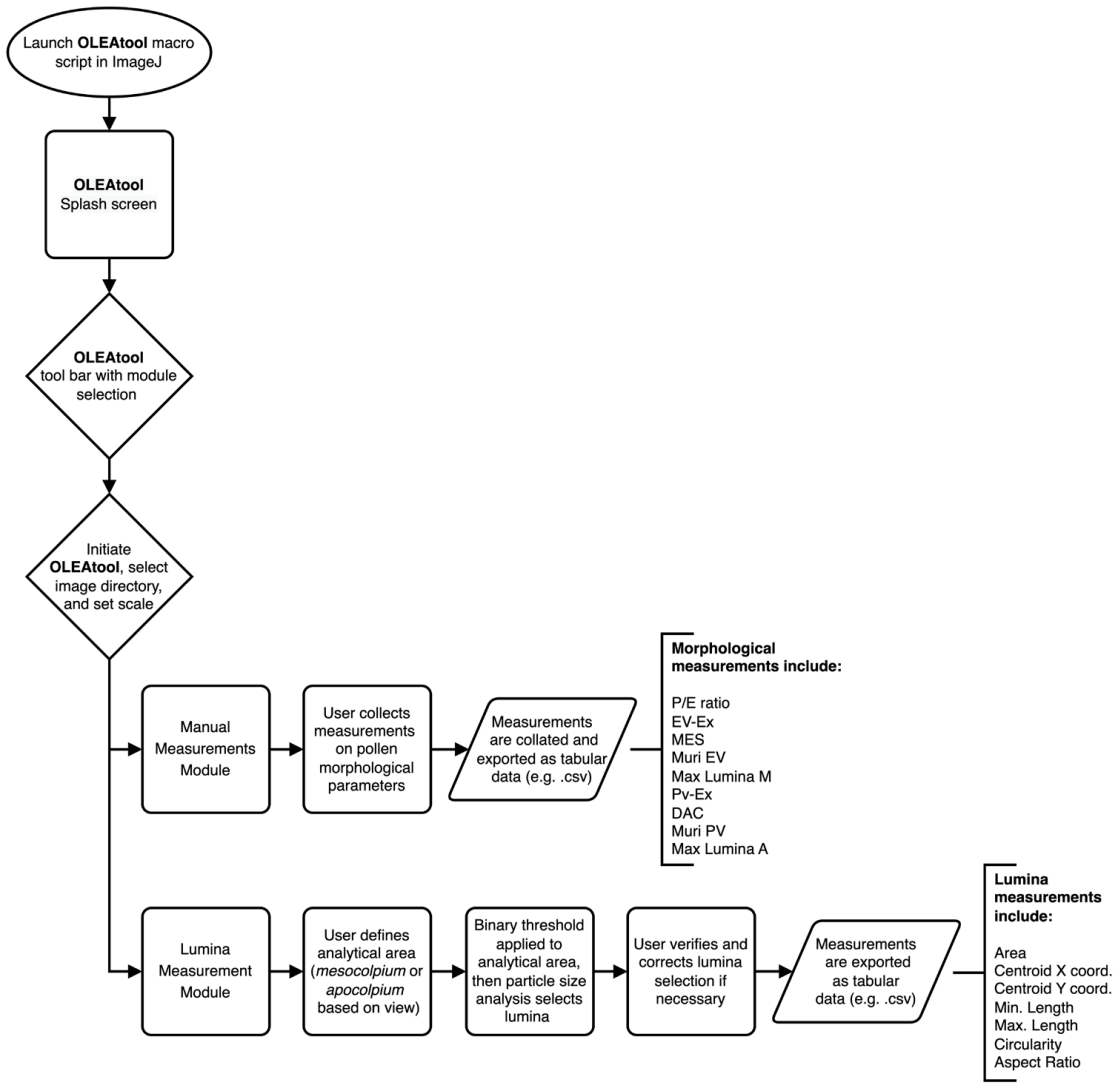
Schematic workflow for OLEAtool. P / E: polar axis and equatorial diameter ratio; EV-Ex: exine thickness in equatorial view; MES: maximum distance between two colpi in mesocolpium; Muri: reticulus thickness; Lumina M: maximum length of lumina in mesocolpium; PV-Ex: exine thickness in polar vision; DAC: distance between the apices of two adjacent colpi; Lumina A: maximum length of lumina in apocolpium.

The schematic workflow for OLEAtool is shown in Figure 2. OLEAtool is built using the ImageJ Macro language (version 1.53k;) to customize and implement a series of standardized tools for measuring pollen grain morphology from high-magnification images collected from camera-mounted light microscopes. Since it operates within ImageJ, OLEAtool is platform independent and is compatible with most common image file formats (e.g., TIFF, JPEG, GIF, BMP, PGM, PNG). OLEAtool includes two modules for measuring pollen morphology: 1) a general module for collecting manual measurements of all morphological parameters ("Manual Measurements Module"), and 2) a semi-automated module for measuring pollen lumina size, shape, and spatial distribution ("Lumina Module"). The operation of each module is described in detail below. Additionally, OLEAtool includes workflows for creating systematic metadata which documents important information about each sample and the data generated through the analysis (e.g., sample number, collection code, polar or equatorial view, analyst, date of analysis). This information is vital for archiving, sharing, and replicating pollen morphology datasets generated through OLEAtool. 

***Manual Measurements Module description.*** Basic operations in OLEAtool are performed using the Manual Measurements Module, which includes linear measurements pollen morphological features in both polar and equatorial views. A user opens an image sequence of a pollen grain and selects the image (or images) that highlights the specific morphological parameter of interest (e.g., *P*, *E*, *EV-Ex*, etc.). For example, if measuring the equatorial diameter ( *E*) of the pollen grain, the user will select an image where the exine is clearly visible and focused. They will draw a line to make the measurement, then click the “E” metric button on the toolbar. This measurement is then collected, and the result is automatically populated within the results table. This process is repeated for all desired measurements in the Manual Measurements Module. See Table 1 for descriptions of each morphological parameter measured in the OLEAtool Manual Measurements Module.

**Table 1.** Description of the parameters measured through the manual and lumina modules.

Module	Metric	Description
**Manual**	P	Polar axis diameter
	E	Equatorial axis diameter
	P/E ratio	Polar axis/equatorial axis ratio
	EV-Ex	Exine thickness in equatorial view
	MES	Maximal distance between colpus in the mesocolpium
	Muri EV	Exine reticulum thickness in mesocolpium
	Max Lumina M	Maximal lumina length in the mesocolpium
	Pv-Ex	Exine thickness in polar view
	DAC	Distance between the apices of two colpi
	Muri PV	Exine reticulum thickness in apocolpium
	Max Lumina A	Maximal lumina length in the apoccolpium
**Lumina**	Area	Area of selected pixels in calibrated units
	Centroid X	The x coordinate of the centroid derived from the center point of the selected lumen
	Centroid Y	The y coordinate of the centroid derived from the center point of the selected lumen
	Min Length	The shortest distance between any two points along the selected lumen
	Max Length	The longest distance between any two points along the selected lumen
	Circularity	Calculated as 4π × area ÷ perimeter ^2^; A value of 1.0 indicates the selected pixels are a perfect circle; As values approach 0.0, the selected pixels are increasingly elongated
	Aspect ratio	Aspect ratio of selected pixels; calculated as the major (primary) axis / minor (secondary) axis of the best fitting ellipse

***Lumina Module description.*** The Lumina Module is an enhance data collection workflow designed to measure a pollen grain’s lumina lengths, locations, and shapes as they are viewed within both the apocolpium and the mesocolpium. See Table 1 for descriptions of each *lumen* parameter measured in the OLEAtool Lumina Module. This module functions by using a user-defined area of interest within the image of a pollen grain to quantify lumina attributes by selecting and measuring them through image thresholding and a particle size analysis procedure. Image thresholds are automatically set but can be fine-tuned by the analyst to highlight as many lumina as possible within the field of view. Particle size analysis is then performed to identify and select all lumina identified through the procedure. Manual adjustment can be made to add or delete misidentified or unidentified lumina. Once the final selection is made, the length, location, and shape of each lumina is measured and compiled into a results table.

This approach offers several key advantages over traditional protocols for measuring pollen lumina in the apocolpium and the mesocolpium. Importantly, this protocol identifies all lumina within the area of interest and measures their maximum and minimum length, allowing a user to quickly identify the largest lumina in each pollen grain, which is commonly used as a distinctive morphological attribute in differentiating between pollen taxa. However, the OLEAtool Lumina Module expands the use of lumina attributes for differentiating pollen morphologies by: 1) constructing the distribution of all lumina maximum lengths within the pollen grain, and 2) collecting additional metrics for each lumen, including shape descriptors and relative centroid coordinates. Eliminating the need to manually measure each lumen within a pollen grain presents new pathways for using multiple metrics to describe all lumina to differentiate between pollen taxa or cultivars.

### Operation

***System requirements.*** OLEAtool is operated using customized macro extensions in ImageJ, and consequently has the same system requirements. ImageJ runs on operating systems that have Java 8 (or later) runtime installed. See installation instructions and examples of system configurations on the ImageJ website ( here). 

***Download and start-up instructions***

1. Download OLEAtool .zip folder from GitHub - see *Software availability* ( Snitker *et al*., 2022)
2. Unzip the folder and place in an easily accessible location, such as the desktop, applications folder, or the home drive.
3. The file structure for photos of pollen used in OLEAtool can be arranged in any manner that is convenient to the user. However, we suggest creating a main folder with an identifying sample number (e.g., sample type, location, or depth) that contains subfolders containing photo sequences for each individual pollen grain that is analyzed. Please note that the main folder name and image file names are extracted and used to create a sample identifier in OLEAtool results table.
4. Open ImageJ and click the “Launch OLEAtool” option from the “More Tools” menu, which can be accessed by clicking the double arrows on the righthand side of the ImageJ toolbar.
5. Click the OLEAtool icon to initiate the program’s start window. A splash screen with the OLEAtool logo will appear on the screen and close. The main menu will then appear, providing the user with a choice to run the manual measurement module or the lumina module in OLEAtool.
6. Detailed and up to date tutorials, demonstration videos, and additional instructions can be found in the OLEAtool folder in GitHub and Zenodo repositories - see *Software availability* ( Snitker *et al*., 2022).

**Figure 3.**
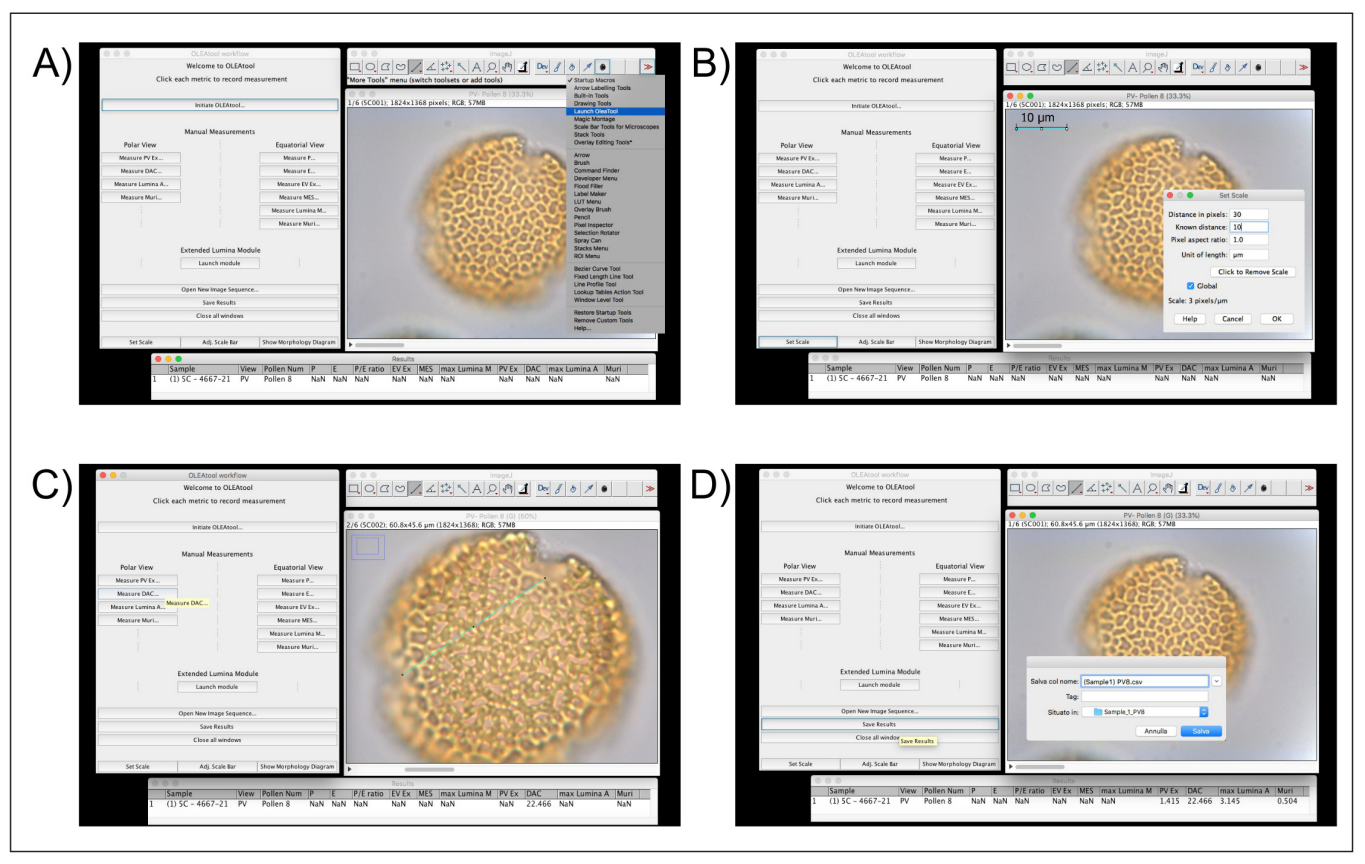
Example of OLEAtool operation in the Manual Measurements Module. Operational steps include **A**) launching OLEAtool from the ImageJ menu; **B**) setting the scale for all measuring operations; **C**) collecting manual measurements by drawing on the image with the line tool and selecting the appropriate measurement to collect; and **D**) saving the output and clearing the images.

***Operating the Manual Measurements Module.*** This module has been created to measure the standard parameters used in *Olea* pollen morphology studies (e.g., Javady & Arzani, 2001; Lanza *et al*., 1996; Messora *et al*., 2017; Ribeiro *et al.*, 2012). The measurements are collected by using a measurement toolbar, which is divided in equatorial (EV) and polar view (PV) parameters. The operational workflow for the Manual Measurements Module is highlighted in Figure 3 and described below:

1. Open the images containing the photos of pollen grains that need to be measured. OLEAtool will extract the metadata from the image and displays them in a results table.
2. Before proceeding with the measurements, it is essential to set the measurement scale and units using the *Set Scale* workflow within OLEAtool. To do so, the software will prompt the user to draw a line of a known length on the current image. Once the line is draw, click [OK], then enter in the known distance of the line in microns (µm). Click [OK]. OLEAtool’s scale is now calibrated.
3. To proceed with the measurements, the user draws a segment on the image and specifies the type of morphological parameter it is measuring, after which the measured value will be immediately reported in the results table.
4. Once all desired measurements are completed, the analyst saves the results table as a tabular dataset (Comma separated values [.csv] or Microsoft Excel file [.xlsx] formats) and can quit the session or open another image sequence to analyze.

**Figure 4.**
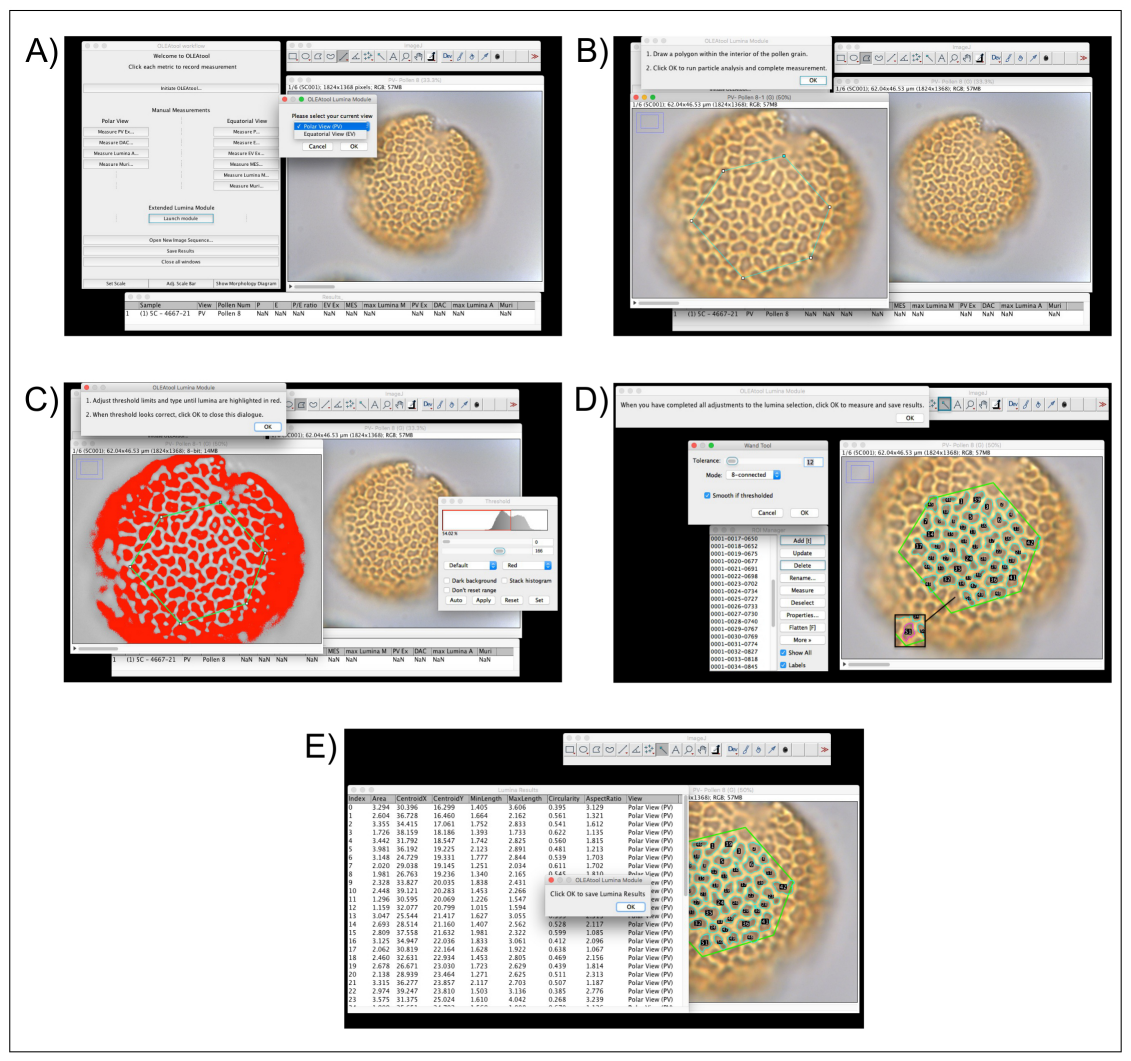
Example of OLEAtool operation in the Lumina Module. Operational steps include **A**) starting the Lumina module and selecting the current view of the pollen grain; **B**) drawing a polygon in the center of the pollen grain to indicate the area of interest; **C**) apply the automated threshold and making any necessary manual adjustments; **D**) running the particle analysis procedure and making any manual adjustments to the selected lumina; and **E**) measuring all selected lumina and saving the results.

***Operating the Lumina Module.*** Exine structure and decoration patterns are key parameters in distinguishing between wild and cultivated *Olea* pollen types. Consequently, the semi-automated components of the Lumina module can efficiently collect multiple measurements of the lumina in the meso- and apocolpium areas. As mentioned previously, this module is expanding the ability for an analyst to evaluate different parameters for each lumen that would otherwise be logistically difficult and very time-consuming if assessed using traditional manual and optical microscopy techniques. The operational workflow for the Lumina Module is highlighted in Figure 4 and described below:

1. After setting the scale, the analyst launches the Lumina Module and specifies the type of view used in the analysis (equatorial or polar view).
2. OLEAtool opens a new window where is possible to choose the image in which the lumina are most visible. The analyst then uses the polygon tool to outline the boundaries of the *mesocolpium* (in equatorial view) or the *apocolpium* (in polar view). All lumina measurements will be take place in area of interest, which is defined by the polygon.
3. OLEAtool then thresholds the image using a black to white color scale. The analyst must either accept the automated threshold or adjust it using the sliders to highlight the most lumina possible. Following this selection, OLEAtool runs a particle size analysis procedure on the threshold image to select the lumina.
4. The lumina identified through the particle analysis are overlayed on the original image and their corresponding selection polygon are listed in the ImageJ region of interest (ROI) viewer. The analyst then verifies that the lumina selection is correct and can add or remove lumina using the ROI viewer. Once the final selections are made, all of the lumina are quantified (i.e., max length, shape, or location).
5. The lumina results can then be exported as tabular data (Common Separated Values [.csv] or Microsoft Excel file [.xlsx] formats), along with the image with the final overlay of selected lumina, which is saved as a JPEG or other supported image format.

## Use Case: Quantifying *Olea* pollen morphology in the Balearic Islands

The main motivation to develop OLEAtool was to increase the number of measurable parameters we could collect, as well as improve the quality and accuracy of *Olea* pollen morphology studies. In this section we present a use case developed from research conducted during the EU-funded OLEA-project (G.A. 895735). The larger study design analysed the similarity/dissimilarity of cultivated and wild olive pollen grains from the Balearic Islands and proposed potential morphotypes that could also be applied to fossil palynological records ( Mercuri *et al.*, 2022; Ricucci, 2022). Here, we highlight a small portion of this work to illustrate the types of data required for using OLEAtool and the process of collecting morphological measurements using the Manual Measurements Module. The data associated with the *Use case* is available in *Underlying data* ( Ricucci *et al*., 2022).

Input for OLEAtool began by collecting *Olea* pollen from olive tree flowers representing different individuals from throughout the Balearic Islands. Samples were then processed using standardized laboratory procedures ( Erdman, 1969). This includes treating each sample with glacial acetic acid to facilitate the release and dehydration of pollen from the anthers. The solution is then filtered, treated with the acetolytic mixture (acetic anhydride and sulfuric acid 9: 1) and boiled (90 ° C for 3–5 minutes). This procedure removes the cytoplasm and the lower layer of the granule wall, so that the outer layer (with diagnostic characters) is more visible. Individual *Olea* pollen were photographed using a light-microscope at 1000x magnification ( Mercuri *et al*., 2022; Ricucci, 2022). The resulting images were saved as JPGs with sample numbers in their file names in preparation for analysis in OLEAtool.

**Figure 5.**
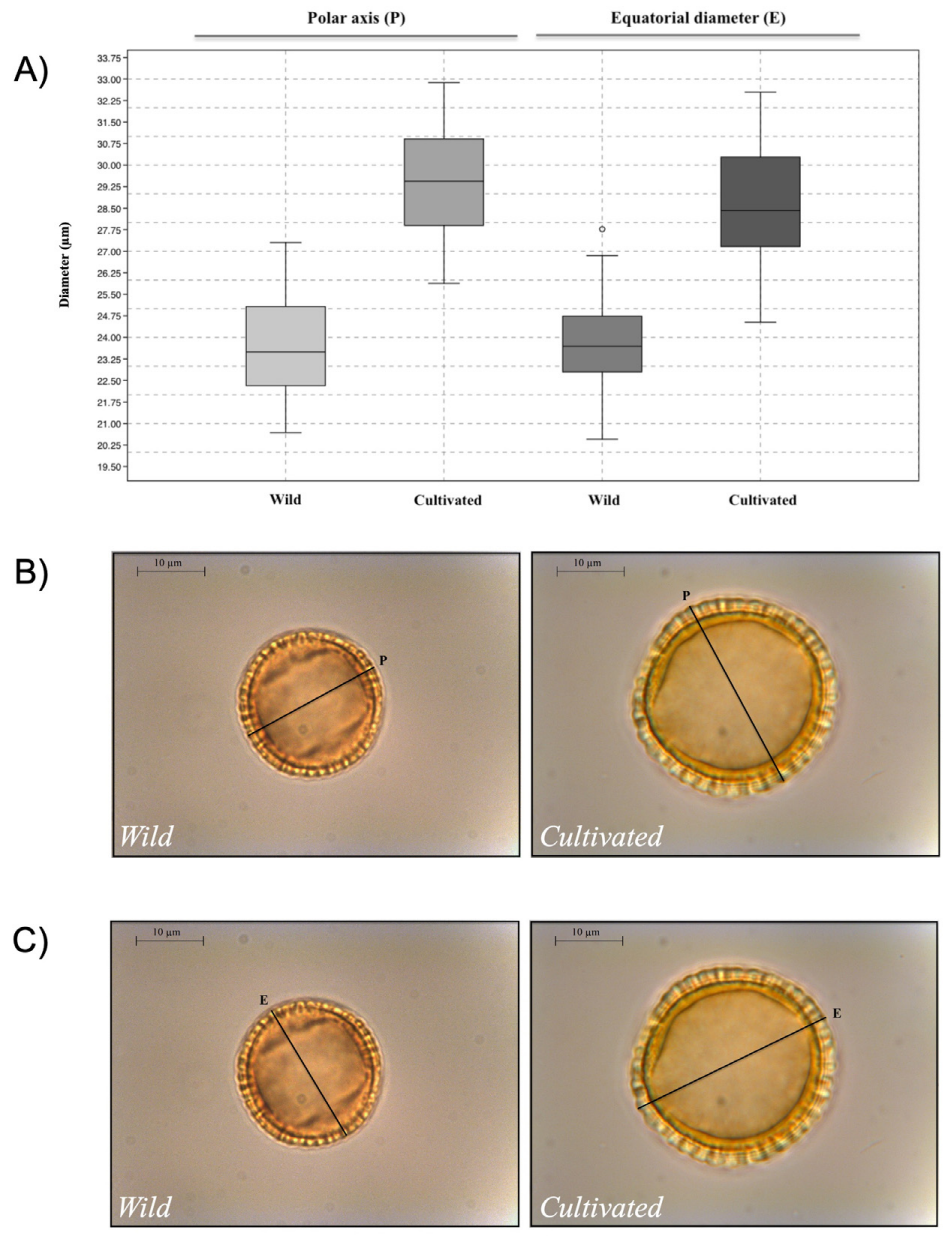
Results from the OLEA-project use study. **A**) Boxplot obtained from the measurements of the polar axis ( *P*) and equatorial diameter ( *E*) parameters in wild and cultivated olive tree samples; **B**) examples of the polar axis ( *P*) measurement in wild and cultivated *Olea* pollen; and **C**) examples of the equatorial diameter ( *E*) measurement in wild and cultivated *Olea* pollen.

In the larger OLEA-project, all possible measurements of *Olea* morphological parameters (see Table 1 and Figure 1) were collected using both the Manual Measurements and Lumina Modules. Here we highlight the results of a subset of measurements made in the Manual Measurements Module and that include the Polar axis ( *P*) and Equatorial diameter ( *E*) collected for wild and cultivated varieties of *Olea* ( Figure 5). Example images of the pollen used to collect these measurements (inputs) and the resulting measurements (outputs), are available via the data archives linked the *Data availably* section below ( Ricucci *et al*., 2022). The mean polar axis measurements for wild varieties were 23.68 ± 1.71µm, while the mean measurements of cultivated varieties slightly higher, with values of 29.41 ± 1.80µm. Similarly, mean equatorial diameters for wild varieties were 23.88 ± 1.48µm and 28.67 ± 2.05µm in pollen corresponding to olive tree cultivars. Basic pollen dimensions accompanied by complementary measurements collected in the larger research project have proven to be key parameters in discriminating group of cultivars ( Messora *et al*., 2017). In this sense, OLEAtool is an important step forward in collecting multiple pollen morphological parameters that when applied, will advance efforts to further distinguish between domestic and wild olive, and among agronomic varieties, though morphological pollen analysis.

## Conclusions

OLEAtool has proven to be a powerful software tool for morphopalynological analysis on *Olea* pollen by increasing the quality, quantity, and speed at which these data can be collected. Specifically, it improves the data collection in the following ways:

1) OLEAtool enhances morphological data collection by increasing the speed, efficiency, replicability of the process by migrating all analyses to a digital platform.
2) The data produced by OLEAtool are standardized, comparable, and reproducible, which can facilitate collaboration and data sharing within the palynological research community.
3) The Lumina Module collects information on maximum lumen length, as well as a suite of other metrics that describe lumina shapes, sizes, and locations within the pollen grain. These new parameters will undoubtably advance the characterization of pollen exine pattern and provide new avenues for morphological analysis of *Olea* pollen.

Forthcoming research will include new statistical approaches to more definitively discriminate between wild and cultivated olive tree pollen, as well as expand the applicability of this work to other sectors, such as identifying modern agronomic varieties of olive trees. The morphotypes generated through this project will be applied to fossil pollen datasets obtained in natural and archaeological site sequences from the Balearic Islands to understand the timing and process of olive cultivation over the last several millennia. Finally, we envision OLEAtool as an initial step in creating more collaboration between palynologists working in the Mediterranean by offering standardized methods for collecting morphological data.

## Ethics and consent

Ethical approval and consent were not required.

## Data Availability

Zenodo: Pollen Images for OLEAtool Manual Measurements Demonstration. https://doi.org/10.5281/zenodo.7412014 ( Ricucci *et al*., 2022). This project contains the following underlying data: 120 pollen images [JPEG format] of wild and cultivated pollen used as input data in demonstration case presented in the text. OLEAtool_example_dataset_manual_measurements.xlsx (all morphological measurements that can be collected in the Manual Measurements Module - see Table 1 - generated as output data from the example pollen images). Data are available under the terms of the Creative Commons Attribution 4.0 International license (CC-BY 4.0).

## Software availability

- Source code available from: https://github.com/gsnitker/OLEAtool
- Archived source code at time of publication: https://doi.org/10.5281/zenodo.7046770 ( Snitker *et al*., 2022)
- License: GPL-3.0-only
