# Supplementary material for: OLEAtool: An open-source software for morphopalynological research in Olea europaea L. pollen
Source: Open Res Eur. 2024 Mar 11;3:29. Originally published 2023 Feb 8. [Version 2] doi: 10.12688/openreseurope.15309.2 (PMC11153994; doi:10.12688/openreseurope.15309.2)
Supplement: Peer review history

# Reviewer report: Reviewer response for version 2

**DOI:** 10.21956/openreseurope.18890.r38852

**Published:** 2024-06-05

**Authors:** Sebastian Pérez-Díaz

**Affiliations:** 1 Universidad de Cantabria, Santander, Cantabria, Spain

I have considered the new version of the manuscript in detail and in my opinion it is acceptable in its present form. The introduction is very interesting and introduces the problem under study. The methods are now better explained and the preliminary results look promising.

Is the rationale for developing the new software tool clearly explained?

Yes

Is the description of the software tool technically sound?

Yes

Are sufficient details of the code, methods and analysis (if applicable) provided to allow replication of the software development and its use by others?

Partly

Is sufficient information provided to allow interpretation of the expected output datasets and any results generated using the tool?

Partly

Reviewer Expertise:

Palynology Paleoenvironment Paleoclimatology Morphometry

I confirm that I have read this submission and believe that I have an appropriate level of expertise to confirm that it is of an acceptable scientific standard.

# Reviewer report: Reviewer response for version 2

**DOI:** 10.21956/openreseurope.18890.r38853

**Published:** 2024-05-03

**Authors:** Sandra Garcés Pastor

**Affiliations:** 1 University of Barcelona, Barcelona, Spain

I think the revision has addressed most of my comments. I only have some minor comment: 

 - Figure 3 and 4: the letters are very small, and it is difficult to read

 - Figure 5: increase the resolution of the table and graph.

 I think this software will be very helpful to separate wild from cultivated species in modern and palaeoecological samples.

Is the rationale for developing the new software tool clearly explained?

Yes

Is the description of the software tool technically sound?

Yes

Are sufficient details of the code, methods and analysis (if applicable) provided to allow replication of the software development and its use by others?

Partly

Is sufficient information provided to allow interpretation of the expected output datasets and any results generated using the tool?

Partly

Reviewer Expertise:

Pollen, eDNA, palaeoecology

I confirm that I have read this submission and believe that I have an appropriate level of expertise to confirm that it is of an acceptable scientific standard.

# Reviewer report: Reviewer response for version 1

**DOI:** 10.21956/openreseurope.16551.r31951

**Published:** 2023-07-26

**Authors:** Sandra Garcés Pastor

**Affiliations:** 1 University of Barcelona, Barcelona, Spain

This article introduces OLEAtool, a software designed to facilitate pollen analysis, enabling researchers to measure and distinguish between various pollen types accurately. The manuscript is well written, thoughtfully structured, and presented in a manner that is both engaging and easy to follow.

 Morphological analysis of pollen is time-consuming, and distinguishing between pollen from the same botanical family can be challenging. Automatic recognition of pollen and spores in fossil pollen samples is a growing trend, allowing to speed the counting and species identification. OLEAtool has the potential to measure and separate many Oleaceae species and varieties that are nowadays difficult to differentiate. The fact that the software allows to analyse several variables at the same time allows a more detailed and standardized measurements, and therefore replicable morphological analyses.  

 The authors' decision to employ open-source software reflects their commitment to upholding the fundamental principles of FAIR and Open Science. This approach not only ensures that the research findings are Findable, Accessible, Interoperable, and Reusable, but also empowers the entire pollen community and citizen scientists to actively participate in the process. This is very important in the context of socio-economic and geographic differences, and benefits society as a whole. 

 I only have some minor comments:

- I had some difficulties installing the OLEAtool software. Download and start-up instructions could also be uploaded in a readme document in GitHub.
- As the other reviewer suggested about the digestion method. I would also add a sentence about the differences that we can expect when using samples that are stored in glycerin or silicone.
- Can this software be extended to other pollen types that are also challenging to identify? Like Brassicaceae? Could you add a sentence about other potential use for other pollen families?
- I wonder if it is possible to propose a repository to upload the measurement data once the research findings are published, as a good practices, so it is more accessible for the scientific community.
- Uploading the pictures is fast, however, it takes a while to realise that all the pictures are uploaded at the same time, and you have to move the bar to go from one to another.
- Some buttons are too small to read all the text. 
- If possible, include a summary of the results obtained using OLEAtool. 

This software, aligned with the Olea-project, will be very helpful in disentangling morphotypes from pollen records, enabling researchers to trace back olive cultivation through time and shed light on the drivers and timing of the *Olea *spread in palaeoecological samples.

Is the rationale for developing the new software tool clearly explained?

Yes

Is the description of the software tool technically sound?

Yes

Are sufficient details of the code, methods and analysis (if applicable) provided to allow replication of the software development and its use by others?

Partly

Is sufficient information provided to allow interpretation of the expected output datasets and any results generated using the tool?

Partly

Reviewer Expertise:

Pollen, eDNA, palaeoecology

I confirm that I have read this submission and believe that I have an appropriate level of expertise to confirm that it is of an acceptable scientific standard, however I have significant reservations, as outlined above.

# Reviewer report: Reviewer response for version 1

**DOI:** 10.21956/openreseurope.16551.r31949

**Published:** 2023-06-12

**Authors:** Sebastian Pérez-Díaz

**Affiliations:** 1 Universidad de Cantabria, Santander, Cantabria, Spain

This article addresses a crucial issue in research on one of the most important species in the Mediterranean, such as Olea, whose situation in the past and expansion remains unclear. To settle this question, it would be very interesting to be able to distinguish between wild and cultivated populations. The work is clear, well planned and structured and with adequate language. It also uses adequate and updated bibliographical references, which gives added value to this work. The use of free software is very important, so that the new methodology is fully accessible to the interested public.

 After reading this work, I have some doubts that perhaps the authors could take into account:

1. Something that raises some doubts is OLEAtool's ability to update the software periodically, either through ImageJ or OLEAtool 's own development. Perhaps the authors could explain this issue in detail.
2. One of the possible weaknesses in the use of different software tools to identify species from the fossil record is related to their conservation. When working with current samples from the current pollen rain, the conservation of pollen grains is generally good, and all the morphometric measurements that are proposed can be very useful. However, when working with samples from the fossil record, it is very frequent that the pollen grains do not present a good conservation condition, since these remains may appear broken, worn, folded, partially corroded, etc. In the case of samples that come from organic archives such as peat bogs or lakes, preservation may be better. But in the case of samples that come from archaeological sites, preservation can be much more complicated. These conservation problems can affect the morphometric measures that determine whether a species is wild or cultivated. Perhaps the authors could explain if this is contemplated in OLEAtool
3. It would have been interesting if the authors provided more data on the collection of samples of wild and cultivated species: sampling area, season of the year, climatic conditions or other parameters that could potentially alter the anatomical structure (fundamentally the size) of the pollen grains. It would also be interesting if the authors provided the pollen extraction method, since there are different methods.

Although the work is still in progress, I believe that it has a good chance of success and may represent an important advance, especially in the determination of wild and cultivated species, and possibly applicable to other species and situations.

Is the rationale for developing the new software tool clearly explained?

Yes

Is the description of the software tool technically sound?

Yes

Are sufficient details of the code, methods and analysis (if applicable) provided to allow replication of the software development and its use by others?

Partly

Is sufficient information provided to allow interpretation of the expected output datasets and any results generated using the tool?

Partly

Reviewer Expertise:

PalynologyPaleoenvironmentPaleoclimatologyMorphometry

I confirm that I have read this submission and believe that I have an appropriate level of expertise to confirm that it is of an acceptable scientific standard, however I have significant reservations, as outlined above.
